# Genomic Analysis of *Aeromonas veronii* C198, a Novel *Mcr-3.41*-Harboring Isolate from a Patient with Septicemia in Thailand

**DOI:** 10.3390/pathogens9121031

**Published:** 2020-12-09

**Authors:** Rujirat Hatrongjit, Anusak Kerdsin, Dan Takeuchi, Thidathip Wongsurawat, Piroon Jenjaroenpun, Peechanika Chopjitt, Parichart Boueroy, Yukihiro Akeda, Shigeyuki Hamada

**Affiliations:** 1 Department of General Sciences, Faculty of Science and Engineering, Chalermphrakiat Sakon Nakhon province campus, Kasetsart University, Sakon Nakhon 47000, Thailand; rujirat.ha@ku.th; 2 Faculty of Public Health, Chalermphrakiat Sakon Nakhon province campus, Kasetsart University, Sakon Nakhon 47000, Thailand; peechanika.c@ku.th (P.C.); parichart.bou@ku.th (P.B.); 3 Research Institute for Microbial Diseases, Osaka University, Osaka 565-0871, Japan; dantake@biken.osaka-u.ac.jp (D.T.); akeda@biken.osaka-u.ac.jp (Y.A.); hamadas@biken.osaka-u.ac.jp (S.H.); 4 Department of Biomedical Informatics, University of Arkansas for Medical Sciences, Little Rock, AR 72205, USA; thidathip@gmail.com (T.W.); piroonj@gmail.com (P.J.); 5 Division of Bioinformatics and Data Management for Research, Department of Research and Development, Faculty of Medicine Siriraj Hospital, Mahidol University, Bangkok 10700, Thailand; 6 Department of Infection Control and Prevention, Graduate School of Medicine, Osaka University, Osaka 565-0871, Japan

**Keywords:** *Aeromonas veronii*, colistin, *mcr*, genome, Thailand

## Abstract

The resistance of Gram-negative bacteria to colistin, mediated by plasmid-borne *mcr* genes, is an emerging public health concern. The complete genome sequence (4.55 Mb) of a clinical isolate of *Aeromonas veronii* biovar *veronii* obtained from a patient with septicemia was determined using short-read and long-read platforms. This isolate (C198) was found to harbor a novel *mcr-3* gene, designated *mcr-3.41*. Isolate C198 revealed adjacent *mcr-3.41* and *mcr-3*-like genes. It contained one chromosome and two plasmids, both of which encoded a RepB replication protein. Other antimicrobial resistance genes, including *bla_cphA3_, bla_OXA-12_, tetA, rsmA,* and adeF, were also present. Isolate C198 was resistant to amoxicillin–clavulanate, ampicillin–sulbactam and tetracycline, and showed intermediate resistance to trimethoprim–sulfamethoxazole. The isolate was susceptible to piperacillin–tazobactam, carbapenem, third-generation cephalosporins, fluoroquinolones, chloramphenicol, and aminoglycosides. Putative virulence genes in the C198 genome encoded type II, III, and VI secretion systems; type IV *Aeromonas* pili; and type I fimbria, flagella, hemagglutinin, aerolysin, and hemolysins. Multilocus sequence typing revealed a novel sequence type (ST), ST720 for C198. Phylogenetic analysis of the single nucleotide polymorphisms in C198 demonstrated that the strain was closely related to *A. veronii* 17ISAe. The present study provides insights into the genomic characteristics of human *A. veronii* isolates.

## 1. Introduction

*Aeromonas veronii* is a Gram-negative bacterium found in a variety of environmental niches, including water, seafood, meat, and vegetables, and occasionally in the feces of healthy individuals [1,2]. It can cause several diseases in humans, including wound infections, pneumonia, hemolytic uremic syndrome, peritonitis, biliary sepsis, and septicemia [2,3]. One study showed that *A. veronii* was more common in patients with acute gastroenteritis (35.7%) than in patients with extra-intestinal infections (5.9%) [4]. A retrospective study of bacteremia caused by monomicrobial *Aeromonas* spp. at a medical center in southern Taiwan from 2004 to 2011 revealed that *A. veronii* (32.7%), *A. dhakensis* (31.4%), *A. caviae* (28.1%), and *A. hydrophila* (6.5%) were the principal disease-causing agents [5].

The growing trend in plasmid-mediated resistance to antimicrobial classes of clinical importance arose from the rapid dissemination of resistance genes in humans and animals. Plasmid-borne antimicrobial resistance determinants have been detected in *Aeromonas* spp. isolated from freshwater, animals, and even humans [6]. The plasmid-borne genes *mcr-1*–*10* confer resistance to colistin, which is administered as a last-line therapeutic intervention for the treatment of Gram-negative bacterial infections [7]. Among the *mcr* genes, *mcr-3* has been disseminated globally in species in both the orders *Enterobacterales* and *Aeromonas*, with the latter serving as a potential reservoir for this gene [8].

Here, we sequenced the complete genome of an *A. veronii* isolate carrying an *mcr-3* gene that was isolated from the blood of a patient with septicemia in Thailand. We also investigated the genomic characteristics, plasmids, antimicrobial resistance, and antimicrobial susceptibility of this isolate.

## 2. Results and Discussion

### 2.1. General Characteristic of A. veronii C198

In 2016, a Gram-negative bacterium was isolated from a blood specimen of a 68-year-old male with septicemia who was admitted to a tertiary hospital in southern Thailand. He was treated with ceftriaxone, and he eventually recovered. Based on the results of a matrix-assisted laser desorption/ionization time-of-flight mass spectrometry (MALDI-TOF MS) analysis, the isolate, which was named strain C198, was determined to be *A. veronii*. Analysis of its genome sequence using average nucleotide identity (ANI) and Kraken2 assigned the isolate to the biovar *veronii*, out of the two *A. veronii* biovars (*veronii* and *sorbria*). The ANI value between strain C198 and the type strain of *A. veronii* CECT 4257^T^ was 96.15%, above the species cut-off level of 95%.

A vast majority (95.4%) of the *Aeromonas* spp. infections in humans are caused by only four species: *A. caviae* (37.26%), *A. dhakensis* (23.49%), *A. veronii* (21.54%), and *A. hydrophila* (13.07%) [1]. Although the global impact of *Aeromonas* in human infections is unknown, a study in California revealed that the annual incidence of *Aeromonas* infections was 10.5 cases per million people [1]. The estimated incidence of *Aeromonas* bacteremia in France was 0.66 cases per million people [9]. Another study showed that the incidence of bacteremia in Taiwan was 76 cases per one million people [10]. In Thailand, *Aeromonas* spp. have been clinically isolated from human cases of bacteremia and peritonitis [11,12,13].

### 2.2. Genome Features

The whole-genome sequence of *A. veronii* C198 is composed of 4,575,001 bp (N_50_ = 4,550,752 bp) with 58.6% GC content. The final genomic assembly had three contigs, one circular chromosome (4,550,752 bp), and two circular plasmids (13,923 and 10,326 bp). The genome was predicted to contain 4223 genes, including 4065 coding sequences (CDS), 11 5S rRNA genes, 10 16S rRNA genes, 10 23S rRNA genes, 123 tRNA genes, and 4 ncRNA genes. There were no clustered regularly interspaced short palindromic repeats. Tekedar et al. (2019) reported a comparison of 41 publicly available *A. veronii* genomes from various sources. Their genome size ranged from 4.28 to 4.95 Mb, and their G + C ratios ranged from 58.1 to 58.9%. Of these 41 genomes, only strain AVNIH1 had a plasmid [2].

The two plasmids of the *A. veronii* C198 isolate are 13,923 and 10,326 bp in length, encoding 15 and 12 proteins, respectively. The larger plasmid contains a *tetA* gene that was predicted to encode the tetracycline efflux MFS transporter, Tet (A). Both plasmids carried genes encoding AAA family ATPases, EamA family transporters, relaxases, Tn3-like element TnAs1 family transposases, integrase domain-containing proteins, type II toxin-antitoxin system RelE/ParE family toxins, cysteine hydrolases, ribbon–helix–helix CopG family proteins, GGDEF domain-containing proteins, sel1 repeat family proteins, and transcriptional regulators. The gene encoding the plasmid replication protein RepB was also present in both plasmids. PlasmidFinder and PLACNETw did not identify the Inc and MOB replicon types, respectively.

### 2.3. Phylogenetic Analysis

**Figure 1:**
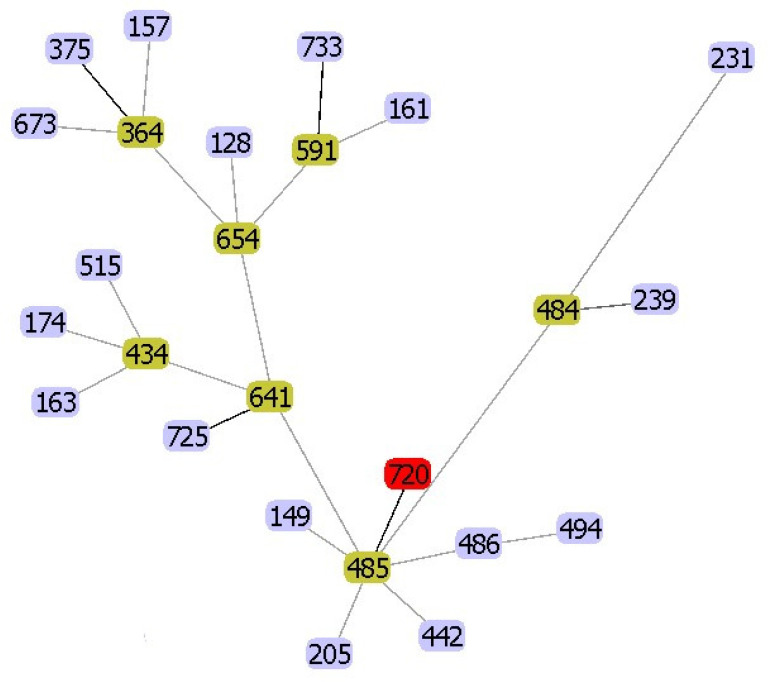
goeBURST analysis of *Aeromonas veronii* C198 (ST720). Other numbers indicate the other sequence types (STs).

Multilocus sequence typing (MLST) revealed that the C198 isolate was a novel sequence type (ST), termed ST720 (*gyrB*: 126, *groL*: 154, *gltA*: 354, *metG*: 16, *ppsA*: 551, *recA*: 374). As shown in Figure 1, ST720 was significantly related to ST485 (*gyrB*: 126, *groL*: 154, *gltA*: 354, *metG*: 16, *ppsA*: 353, and *recA*: 374), an isolate obtained from seafood in China in 2014. There was a single locus variant in *ppsA* between these two sequence types (STs).

**Figure 2:**
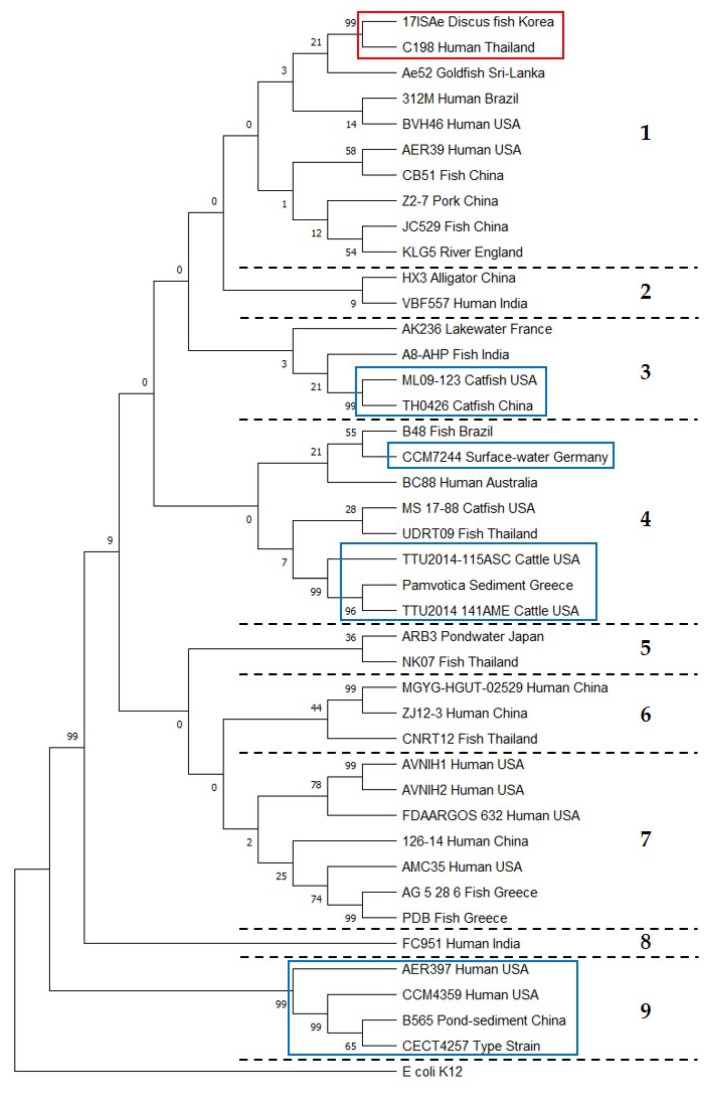
Whole-genome phylogeny analysis of *A. veronii*. A phylogenetic tree was generated using REALPHY and visualized with MEGA-X software using the neighbor-joining method. Nine major clusters are shown, separated by dashed lines and numbers. Blue boxes indicate the four subgroups described by Tekedar et al. (2019), and the red box indicates our isolate and related isolates. *Escherichia coli* K12 was used as an outgroup.

The single nucleotide polymorphism (SNP) phylogeny demonstrated that C198 was closely related to a fish isolate (17ISAe) in Korea (Figure 2). There are four highly conserved genetic subgroups for this: (1) USA dairy cattle isolates and an isolate from Pamvotida Lake, Greece; (2) strain ML09-123 (USA) and strain TH0426 (catfish isolate) from China; (3) human isolates (strains CECT4257^T^, CCM4359, and AER397) from the USA and a sediment isolate from China (B565); and (4) surface water isolates from the USA and Germany [2]. As shown in Figure 2, our phylogenetic analysis revealed nine clusters represented by the following strains: (1) C198 (our isolate); (2) China HX3; (3) USA ML09-123, genetic subgroup 2 described by Tekedar et al. (2019); (4) Greece pamvotica, genetic subgroup 1 of Tekedar et al. (2019); (5) Thailand NK07; (6) China ZJ12-3; (7) USA AVNIH1; (8) India FC951; and (9) a type strain CECT4257^T^ of genetic subgroup 3 [2]. Interestingly, human *A. veronii* isolates are distributed throughout the phylogenetic tree, which means they are genetically diverse.

### 2.4. Antimicrobial Susceptibility and Resistance Genes

Antimicrobial susceptibility testing revealed that *A. veronii* C198 was resistant to amoxicillin–clavulanate, ampicillin–sulbactam, and tetracycline, but showed intermediate resistance to trimethoprim–sulfamethoxazole (Table 1). It was susceptible to piperacillin–tazobactam, carbapenem, third-generation cephalosporins, fluoroquinolones, chloramphenicol, and aminoglycosides (Table 1). Some studies have reported that *A. veronii* is usually resistant to amoxicillin–clavulanate and is susceptible to carbapenem, third- and fourth-generation cephalosporins, aminoglycosides, monobactam, and fluoroquinolones [1,8,14,15]. The minimal inhibitory concentration (MIC) of colistin for our isolate was 2 µg/mL, which could not be interpreted as susceptible, intermediate, or resistant by either the Clinical Laboratory Standards Institute (CLSI) (M45) or EUCAST guidelines due to the lack of a clinical breakpoint for colistin in *Aeromonas* spp. However, this MIC was similar to those of *A. veronii* strains 172, Z2-7, and w55, which were also 2 µg/mL [8,14,15].

Whole-genome sequencing revealed the presence of *mcr-3,* *bla_cphA3_, bla_OXA-12_, tetA, rsmA*, and adeF genes, which are likely responsible for the observed resistance to various antimicrobials (Table 1 and Table 2). Most of these resistance genes are located on the chromosome, except for *tetA*, which is located on the larger (13,923 bp) plasmid. The presence of *bla_cphA3_* and bla_OXA-12_ may contribute to the observed resistance to ampicillin–sulbactam and amoxicillin–clavulanate. However, these genes do not confer resistance to piperacillin–tazobactam, third-generation cephalosporins, or carbapenem, because our isolate was susceptible to these antimicrobials. The tetA gene likely contributed to tetracycline resistance, and mcr-3.3 may be responsible for the observed MIC of colistin (2 μg/mL).

Comparison of the antimicrobial resistance genes in our isolate to those in other A. veronii isolates revealed the presence of mcr-3 in the genomes of isolates from China, Korea, and the USA (Table 2). The pattern of antimicrobial resistance genes in C198 was similar to those in strains 126-14 and HX3, which were isolated from humans and alligators, respectively, in China; only the tetA gene was different. Almost all isolates contained bla_cphA_, bla_OXA-12_, adeF, rsmA, and EF-Tu mutant (R234F). This suggests that these genes are conserved in A. veronii. The current study revealed that mcr-3 genes were present in some, but not all, A. veronii genomes (Table 2), indicating that A. veronii does not inherently carry the mcr-3 gene. However, A. veronii may be a reservoir for the dissemination of mcr-3 genes to other bacteria, as previously reported [14,16].

**Figure 3:**
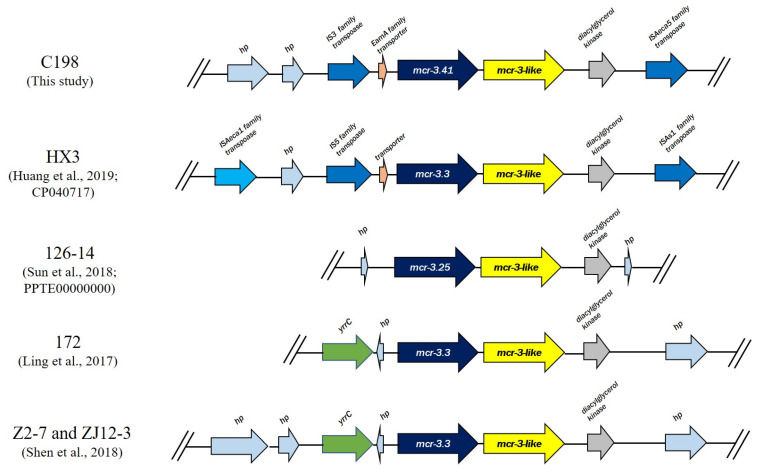
Comparison of the genetic organization surrounding the *mcr-3* and *mcr-3*-like genes in five *A. veronii* isolates. The schematic shows the genes flanking the *mcr-3* and *mcr-3*-like genes in these isolates.

The genetic organization surrounding *mcr-3* in the C198 isolate was examined. As shown in Figure 3, the *mcr-3* and *mcr-3*-like genes were adjacent to each other. Similar findings have been reported for two adjacent *mcr-3* genes in other *Aeromonas* spp., including *A. veronii* isolates [8,14]. Among the previously evaluated genes, only *mcr-3.3* conferred colistin resistance, whereas *mcr-3*-like genes did not affect the MIC of colistin [14]. Both the *mcr-3* and *mcr-3*-like genes of the C198 isolate were located on the chromosome and were flanked by diacylglycerol kinase (*dgk*) and ISAeca5 family transposase genes downstream, and an EamA family transporter and the IS*3* family transposase genes upstream (Figure 3). This is similar to the gene arrangement observed in isolate HX3. A common gene found in all *mcr-3*-harboring *A. veronii* isolates is *dgk*, which is located downstream of the *mcr-3*-like gene in C198 (Figure 3).

**Figure 4:**
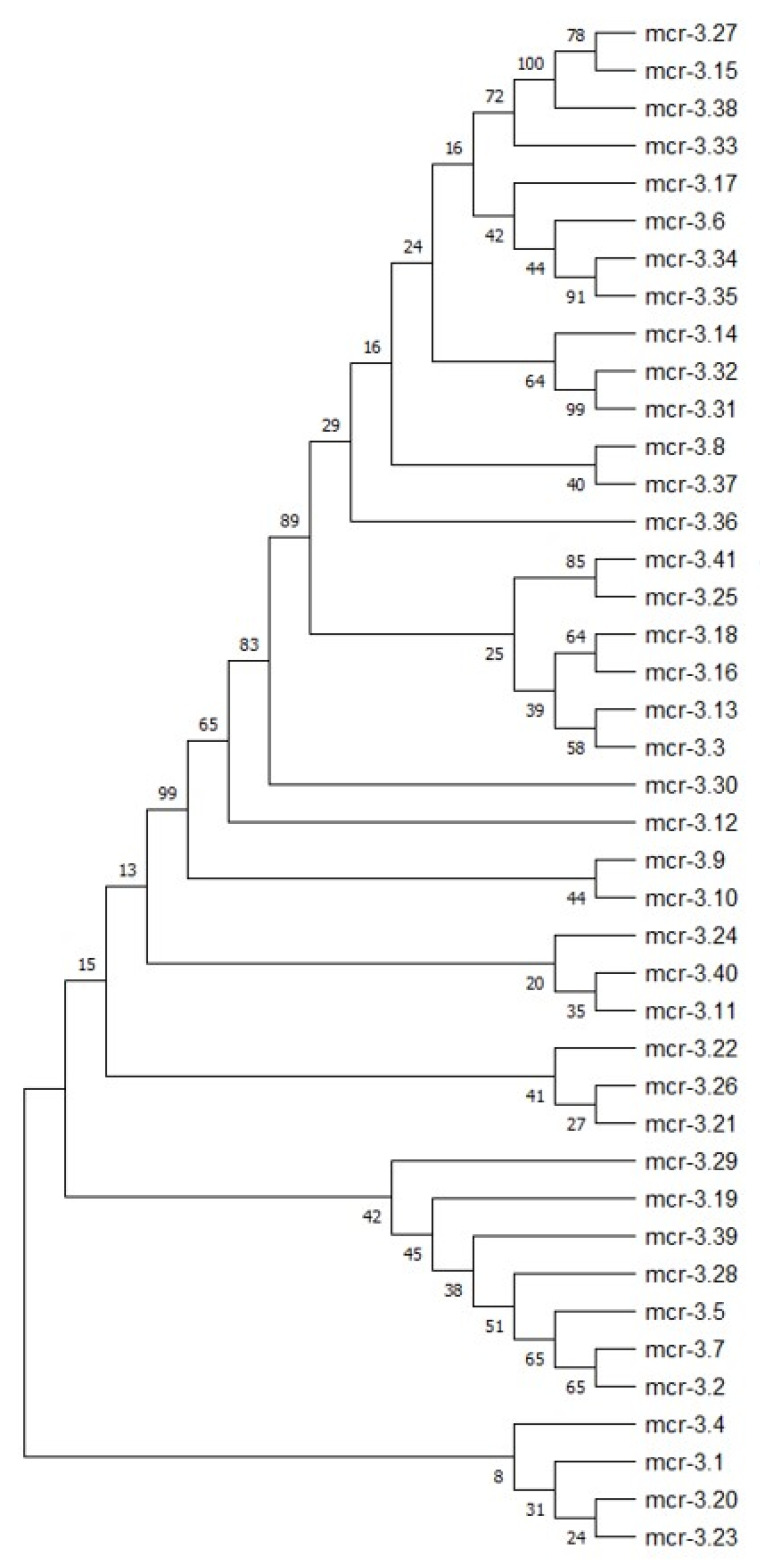
Unrooted neighbor-joining tree based on amino acid sequences of all MCR-3 like variants described. The phylogenetic tree was generated and visualized with MEGA-X software using the neighbor-joining method with 500 bootstraps after applying the Poisson model.

The *mcr-3* variant in our C198 isolate was analyzed using ResFinder and CARD, and these two tools identified different variants: it was identified as *mcr-3.25* by ResFinder and as *mcr-3.3* by CARD. Collectively, the GenBank database then assigned our *mcr-3* variant as a novel *mcr*, named *mcr-3.41* (accession no. MBA2799562.1). The deduced amino-acid sequence of MCR-3.41 (*A. veronii* C198) showed 99.81, 99.44, and 95.55% identity to MCR-3.25 (*A. veronii* 126-14), MCR-3.3 (*A. veronii* 172), and MCR-3.4 (*E. coli* ECCTRSRTH07), respectively. MCR-3.41 differs from MCR-3.25 in one amino acid position (A154T) (Supplementary File S1). All of these data indicated that the MCR-3.41 was more closely related to MCR-3.25 than to MCR-3.3. The phylogenetic tree of all mcr-3 protein sequences is shown in Figure 4. Our novel mcr-3.41 was clustered with mcr-3.25 according to the alignment analysis result as described above. An analysis of the *mcr-3*-like genes among strains C198, 172, and HX3 demonstrated 100% identity among C198, 126-14, and HX3, and 99.81% identity between C198 and 172 (Supplementary File S1).

### 2.5. Virulence Factors

The virulence genes detected in the C198 isolate included genes encoding type II, III, and VI secretion systems, tap type IV pili, type I fimbria, flagella, hemagglutinin, aerolysin, and hemolysins (Table 1). *A. veronii* possesses a type II secretion system that exports hydrolytic enzymes, hemolysin, and aerolysin [1,2]. Type VI systems are known to inject protein effectors, such as G repeat proteins (VgrG) and hemolysin-co-regulated protein (Hcp), directly into the cytosol of the target cell [1]. However, another study reported that seven of nine human *A. veronii* isolates lack a type III system, indicating that specific system may not be crucial for its virulence in mammals [2].

Hemolysins, including aerolysin, are capable of forming pores in the cell membrane, leading to the osmotic lysis of target cells [1]. In addition, aerolysins may alter the permeability of blood cells and other eukaryotic cells, resulting in cell lysis [17]. Tap type IV pili, type I fimbria, flagella, and hemagglutinin are involved in host cell adhesion [1,17].

The pathogenesis of *A. veronii* infection is complex and is an area of active investigation. A recent study identified several virulence factors that were positively correlated with the pathogenicity of *A. veronii* [18]. These virulence genes contribute significantly to the development of infections, and *A. veronii* isolates carrying more virulence genes were more virulent in mice [17,18].

## 3. Materials and Methods 

### 3.1. Bacterial Strain 

A laboratory-based surveillance program for the detection of emerging species of antimicrobial-resistant bacteria in 11 hospitals (Sakon Nakhon, Nakhon Phanom, Surin, Udonthani, Mukdahan, Bueng Kan, Chumporn, Surat Thani, Tak, Phayao, and Bangkok) within representative provinces in Thailand has been ongoing since 2016. The *A. veronii* strain described in this study was isolated from the blood of a patient with septicemia admitted to a tertiary hospital in southern Thailand. 

### 3.2. Antimicrobial Susceptibility Testing

*A. veronii* C198 cells were incubated on blood agar for 24 h at 37 °C. Identification was carried out using matrix-assisted laser desorption/ionization time-of-flight (MALDI-TOF) mass spectrometry (MALDI Sepsityper; Bruker Daltonics, Bremen, Germany). Antimicrobial susceptibility of *Aeromonas* spp. was assessed with the broth microdilution method using 17 antimicrobial agents (amoxicillin–clavulanate, ampicillin–sulbactam, piperacillin–tazobactam, cefepime, cefotaxime, cefoxitin, ceftazidime, ceftriaxone, ertapenem, meropenem, imipenem, aztreonam, gentamicin, amikacin, tetracycline, ciprofloxacin, levofloxacin, trimethoprim–sulfamethoxazole, chloramphenicol, and colistin) in accordance with the 2016 Clinical Laboratory Standards Institute (CLSI) guidelines [19]. The antimicrobial susceptibility profile (resistant, intermediate, or susceptible) was interpreted according to the CLSI-M45-A2 (2016) guidelines [19].

### 3.3. Whole-Genome Sequencing

Bacterial DNA was extracted using the ZymoBIOMICS DNA Kit (Zymo Research, Irvine, CA, USA) and sequenced on the Oxford Nanopore Technologies (ONT) and Illumina MiSeq platforms. The rapid barcoding protocol was followed for ONT-based DNA sequencing using the SQK-RBK004 kit without selecting DNA size to preserve plasmid DNA. The libraries were sequenced using a single R9.4.1/FLO-MIN106 flow cell on a MinION Mk1B sequencer. The raw data were demultiplexed using Guppy v3.4.5 (Oxford Nanopore Technologies (ONT), Oxford, UK), specifying the high-accuracy model (-c dna_r9.4.1_450 bps_hac.cfg). The ONT adapters were trimmed using Porechop v0.2.4 (https://github.com/rrwick/Porechop). Quality control for the ONT reads was performed using NanoPlot v1.28.1 (https://github.com/wdecoster/NanoPlot).

A sequencing library was generated using the NEBNext Ultra II DNA Library Prep Kit for Illumina (New England Biolabs, Ipswich, MA, USA) according to the manufacturer’s recommendations. We applied Fastp v0.19.5 [20] for quality filtering of Illumina reads. Adapters were trimmed using Skewer v0.2.2 [21]. Quality checking of the reads was performed using FastQC v0.11.8 (https://www.bioinformatics.babraham.ac.uk/ projects/fastqc/). Hybrid assemblies of ONT and Illumina data were generated using Unicycler v0.4.8 [22], and the quality of the genome sequences was checked using QUAST v5.0.2 [23]. The circular DNA structures of the bacterial chromosome and This is a free program. No company owns. We used it via website, so, I added the reference is enough.plasmids were computationally produced using Unicycler software. Genome sequences were submitted to the NCBI Prokaryotic Genome Annotation Pipeline v4.12 for validation. Default parameters were used for all software, unless otherwise specified.

### 3.4. Analysis of the Whole-Genome Sequence

The (MALDI-TOF) mass spectrometry species determination was confirmed through ANI [24] and Kraken [25] using the whole-genome sequence. *A. veronii* CECT4257^T^ (Type strain) was used as a reference genome for the ANI comparisons. Antimicrobial resistance genes were identified using ResFinder 4.0 (https://cge.cbs.dtu.dk/services/ResFinder/) and CARD (https://card.mcmaster.ca/home) [26,27]. Virulence genes were identified using the VFDB database [28]. Plasmid type was determined using PlasmidFinder [29] and PLACNETw [30].

Multilocus sequence typing (MLST) analysis to determine the STs of *Aeromonas* spp. was performed using PubMLST (https://pubmlst.org/aeromonas/). A Google BURST analysis of the STs was performed using PHYLOViZ 2.0 [31]. Genomic sequences were compared using a reference genome-based SNP strategy using REALPHY [32]. A phylogenetic tree was constructed from the REALPHY data using MEGA-X with the neighbor-joining method (500 bootstrap replicates) by applying the Tamura three-parameter model [33]. The reference genomes of 40 isolates of *A. veronii* (isolated from humans (*n* = 16), animals (*n* = 18), and external environments (*n* = 6) were downloaded from GenBank for the REALPHY analysis (Table 2). In the case of phylogenetic analysis of all mcr-3 proteins, the tree was constructed using MEGA-X via the neighbor-joining method with 500 bootstrap replicates by applying the Poisson model [33]. The mcr-3 protein sequences used in this study are shown in Supplementary File S2.

### 3.5. Accession Numbers

The complete sequence was submitted to GenBank under the BioProject accession number: PRJNA525849, BioSample accession number: SAMN15587301, and accession number: JACEGL000000000.

### 3.6. Ethics

The Human Research Ethics Committee Office of Osaka University reviewed this protocol and approved this study. The ethics approval number was 14468-5. This study was conducted according to the principles of the Declaration of Helsinki.

## 4. Conclusions

This study revealed the clinical isolate *A. veronii* obtained from a patient with septicemia contained adjacent *mcr-3* and *mcr-3*-like genes. In addition to these, other antimicrobial resistance genes and virulence genes were also present. Phylogenetic analysis demonstrated that the strain was closely related to the *A. veronii* strain obtained from a discus fish. Combining ONT and Illumina sequencing data provided insights into the genomic characteristics of human *A. veronii* isolates.

## Figures and Tables

**Table 1:** Characteristics of the *A. veronii* C198 isolate used in this study.

Characteristics	*A. veronii* C198
Sequence Type	ST720
Antimicrobial susceptibility (μg/mL) *	
Amoxicillin–clavulanic acid	32/16 (R)
Ampicillin–sulbactam	32/16 (R)
Piperacillin–tazobactam	2/4 (S)
Cefepime	0.25 (S)
Cefotaxime	0.25 (S)
Cefoxitin	1 (S)
Ceftazidime	0.5 (S)
Ertapenem	0.5 (S)
Imipenem	0.125 (S)
Meropenem	0.125 (S)
Amikacin	4 (S)
Gentamicin	1 (S)
Tetracycline	32 (R)
Ciprofloxacin	0.25 (S)
Levofloxacin	0.25 (S)
Chloramphenicol	2 (S)
Trimethoprim–sulfamethoxazole	2/38 (S)
Colistin	2 (ND **)
Antimicrobial and resistance genes	
*β*-lactam	*bla_cphA3_*
	*bla_OXA-12_*
Tetracycline	*tetA*
Colistin	*mcr-3*
Fluoroquinolone and tetracycline	*adeF*
Fluoroquinolone, diaminopyrimidine, and phenicol	*rsmA*
elfamycin	EF-Tu (R234F) ^#^
Virulence genes	
Adherence	Flagella
	Mannose-sensitive hemagglutinin
	Tap type IV pili
	Type I fimbria
Secretion system	Type II secretion system
	Type III secretion system
	Type VI secretion system
Toxins	Aerolysin
	Hemolysin III
	Hemolysin, HlyA
	Thermostable hemolysin

R: resistant, S: susceptible, I: intermediate. * Susceptibility to antimicrobials was performed using broth microdilution. ND **: no clinical breakpoint for colistin in *Aeromonas* spp. by either the Clinical Laboratory Standards Institute (CLSI) (M45) or EUCAST guidelines. ^#^ Substitution of amino acid at position 234 in EF-Tu.

**Table 2:** Comparison of antimicrobial resistance genes in *A. veronii* isolates from various specimens.

Strain	Accession No.	Source	Country	*MCR*	*cphA*	*OXA-12*	*OXA-21*	*TEM*	*SHV*	*AAC(6’)*	*AAC(3)*	*aadA*	*APH(3’)-Ia*	*APH(6)-Id*	*qnr*	*tet*	*mphA*	*floR*	*dfrA*	*sul*	*arr-3*	*cat*	*adeF*	*rsmA*	*qacEdelta1*	EF-Tu Mutant
C198	JACEGL000000000	Human	Thailand	3.41	A3	+										A							+	+		R234F
126-14	PPTE00000000	Human	China	3.25	A3	+										D							+	+		R234F
MGYG-HGUT-02529	CABMOE000000000	Human	China	3.3	A3	+	+			IIa		A16				A			A15	1		B3	+	+	+	R234F
ZJ12-3	UETM00000000	Human	China	3.3	A3	+	+			IIa		A16				A			A15	1		B3	+	+	+	R234F
AVNIH1	CP014774	Human	USA		A3	+			134	Iic, Ib10	IIg	A2			B4	D, E	+	+	A12	1, 2			+	+	+	R234F
AVNIH2	LRBO00000000	Human	USA		A3	+										E							+	+		R234F
AER39	AGWT00000000	Human	USA		A3	+																	+	+		R234F
AMC35	AGWW00000000	Human	USA		A3	+																	+	+		R234F
BVH46	NKWS01000000	Human	USA		A3	+																	+	+		R234F
FDAARGOS_632	CP044060	Human	USA		A3	+																	+	+		R234F
AER397	AGWV00000000	Human	USA		A6	+																	+	+		R234F
CCM4359	MRZR00000000	Human	USA		A6	+																	+	+		
CECT4257^T^	CDDK00000000	Human	USA		A6	+																	+	+		
FC951	PKSR00000000	Human	India		A3	+																	+	+		R234F
VBF557	LXJN00000000	Human	India		A3																					R234F
312M	RHDQ00000000	Human	Brazil		A3	+																	+	+		R234F
BC88	CAAKNH000000000	Human	Australia		A7	+																	+	+		R234F
UDRT09	JAAQQM000000000	Fish	Thailand		A3	+		116							S2	E							+	+		R234F
CNRT12	JAAQQN000000000	Fish	Thailand		A3	+																	+	+		R234F
NK07	JAAQQQ000000000	Fish	Thailand		A3	+						A2				A	+		A12	1			+	+	+	R234F
TH0426	CP012504	Catfish	China		A3	+																	+	+		R234F
CB51	CP015448	Fish	China		A3	+																		+		R234F
JC529	CP058912	Fish	China		A3	+																	+	+		R234F
A8-AHP	CP046407	Fish	India		A3	+							+										+	+		R234F
Ae52	BDGY00000000	Goldfish	Sri-Lanka		A3	+						A2	+	+		A, D	+		A12	1		II	+	+	+	
AG-5.28.6	NNSE00000000	Fish	Greece		A3	+																	+	+		
PDB	NMUS00000000	Fish	Greece		A3	+																	+	+		
B48	WSFS00000000	Fish	Brazil		A3	+																	+	+		R234F
ML09-123	PPUW00000000	Catfish	USA			+																	+	+		R234F
MS-17-88	RAWX00000000	Catfish	USA	3, 7.1	A3	+										D		+	A3			B7	+	+		R234F
TTU2014-141AME	LKKD00000000	Cattle	USA		A3	+										E							+	+		
TTU2014-115ASC	LKJS00000000	Cattle	USA		A3	+																	+	+		R234F
Z2-7	UETI00000000	Pork	China	3.3	A3	+										D			A15	1			+	+	+	R234F
HX3	CP040717	Alligator	China	3.3	A3	+										D							+	+		R234F
17ISAe	CP028133	Discus fish	Korea	3.6, 3.8	A3	+				Ib-cr						A			A1	1	+		+	+	+	R234F
CCM7244	MRZQ00000000	Surface water	Germany		A6	+																	+	+		R234F
AK236	NKXF00000000	Lake water	France		A3	+																	+	+		
ARB3	JRBE00000000	Pond water	Japan		A6	+																	+	+		
KLG5	CAAKNL000000000	River	England		A3	+																	+	+		R234F
B565	NC_015424	Sediment	China		A6	+																	+	+		R234F
Pamvotica	MRUI00000000	Sediment	Greece		A3	+																	+	+		R234F

+: means present; blank means absent.

## Supplementary Materials

The following are available online at https://www.mdpi.com/2076-0817/9/12/1031/s1, File S1: Sequence alignment of *mcr-3* between our isolate (C198) and others; File S2: The mcr-3 protein sequences used for phylogenetic tree construction.
