# Supplementary material for: Genomic Analysis of Aeromonas veronii C198, a Novel Mcr-3.41-Harboring Isolate from a Patient with Septicemia in Thailand
Source: Pathogens. 2020 Dec 9;9(12):1031. doi: 10.3390/pathogens9121031 (PMC7763265; doi:10.3390/pathogens9121031)
Supplement: Supplementary file 1 [file pathogens-09-01031-s001.zip › Supplementary files/Supplementary file 1-Sequence alignment of mcr-3 between our isolate (C198) and others.docx]

**Pairwise alignment of MCR-3.25 and a novel MCR-4.1 between strains 126-14 and C198**

C198_mcr-3.41 MPSLIKIKIVPLMFFLALYFAFMLNWRGVLHFYEILYKLEDFKFGFAISLPILLVAALNF 60

126-14_mcr-3.25 MPSLIKIKIVPLMFFLALYFAFMLNWRGVLHFYEILYKLEDFKFGFAISLPILLVAALNF 60

************************************************************

C198_mcr-3.41 VFVPFSIRYLIKPFFALLIALSAIVSYTMMKYRVLFDQNMIQNIFETNQNEALAYLSLPI 120

126-14_mcr-3.25 VFVPFSIRYLIKPFFALLIALSAIVSYTMMKYRVLFDQNMIQNIFETNQNEALAYLSLPI 120

************************************************************

C198_mcr-3.41 IGWVTIAGFIPAILLFFVEIEYEEKWFKGILTR**T**LSMFASLIVIAVIAALYYQDYVSVGR 180

126-14_mcr-3.25 IGWVTIAGFIPAILLFFVEIEYEEKWFKGILTR**A**LSMFASLIVIAVIAALYYQDYVSVGR 180

*********************************:**************************

C198_mcr-3.41 NNSNLQREIVPANFVNSTVKYVYNRYLAEPIPFTTLGDDAKRDTNQSKPTLMFLVVGETA 240

126-14_mcr-3.25 NNSNLQREIVPANFVNSTVKYVYNRYLAEPIPFTTLGDDAKRDTNQSKPTLMFLVVGETA 240

************************************************************

C198_mcr-3.41 RGKNFSMNGYEKDTNPFTSKSGGVISFNDVRSCGTATAVSVPCMFSNMGRKEFDDNLARN 300

126-14_mcr-3.25 RGKNFSMNGYEKDTNPFTSKSGGVISFNDVRSCGTATAVSVPCMFSNMGRKEFDDNLARN 300

************************************************************

C198_mcr-3.41 SEGLLDVLQKTGVSIFWKENDGGCKGVCDRVPNIEIKPKDYPKFCDKNTCYDEVVLQELD 360

126-14_mcr-3.25 SEGLLDVLQKTGVSIFWKENDGGCKGVCDRVPNIEIKPKDYPKFCDKNTCYDEVVLQELD 360

************************************************************

C198_mcr-3.41 SEIAQMKGDKLVGFHLIGSHGPTYYKRYPDAHRQFTPDCPRSDIENCTDEELTNTYDNTI 420

126-14_mcr-3.25 SEIAQMKGDKLVGFHLIGSHGPTYYKRYPDAHRQFTPDCPRSDIENCTDEELTNTYDNTI 420

************************************************************

C198_mcr-3.41 RYTDFVIGEMIAKLKTYEDKYNTALLYVSDHGESLGALGLYLHGTPYKFAPDDQTRVPMQ 480

126-14_mcr-3.25 RYTDFVIGEMIAKLKTYEDKYNTALLYVSDHGESLGALGLYLHGTPYKFAPDDQTRVPMQ 480

************************************************************

C198_mcr-3.41 VWMSPGFITEKGMNMECLQKNAAANRYSHDNIFSSVLGIWDVKTAIYEQELDIFKQCRNN 540

126-14_mcr-3.25 VWMSPGFITEKGMNMECLQKNAAANRYSHDNIFSSVLGIWDVKTAIYEQELDIFKQCRNN 540

************************************************************

**Pairwise alignment of MCR-3.3 and a novel MCR-4.1 between strains 172 and C198**

C198_mcr-3.41 MPSLIKIKIVPLMFFLALYFAFMLNWRGVLHFYEILYKLEDFKFGFAISLPILLVAALNF 60

172_mcr-3.3 MPSLIKIKIVPLMFFLALYFAFMLNWRGVLHFYEILYKLEDFKFGFAISLPILLVAALNF 60

************************************************************

C198_mcr-3.41 VFVPFSIRYLIKPFFALLIALSAIVSYTMMKYRVLFDQNMIQNIFETNQNEALAYLSLPI 120

172_mcr-3.3 VFVPFSIRYLIKPFFALLIALSAIVSYTMMKYRVLFDQNMIQNIFETNQNEALAYLSLPI 120

************************************************************

C198_mcr-3.41 IGWVTIAGFIPAILLFFVEIEYEEKWFKGILTR**T**LSMFASLIVIAVIAALYYQDYVSVGR 180

172_mcr-3.3 IGWVTIAGFIPAILLFFVEIEYEEKWFKGILTR**A**LSMFASLIVIAVIAALYYQDYVSVGR 180

*********************************:**************************

C198_mcr-3.41 NNSNLQREIVPANFVNSTVKYVYNRYLAEPIPFTTLGDDAKRDTNQSKPTLMFLVVGETA 240

172_mcr-3.3 NNSNLQREIVPANFVNSTVKYVYNRYLAEPIPFTTLGDDAKRDTNQSKPTLMFLVVGETA 240

************************************************************

C198_mcr-3.41 RGKNFSMNGYEKDTNPFTSKSGGVISFNDVRSCGTATAVSVPCMFSNMGRKEFDDNLARN 300

172_mcr-3.3 RGKNFSMNGYEKDTNPFTSKSGGVISFNDVRSCGTATAVSVPCMFSNMGRKEFDDNLARN 300

************************************************************

C198_mcr-3.41 SEGLLDVLQKTGVSIFWKENDGGCKGVCDRVPNIEIKPKDYPKFCDKNTCYDEVVLQELD 360

172_mcr-3.3 SEGLLDVLQKTGVSIFWKENDGGCKGVCDRVPNIEIKPKDYPKFCDKNTCYDEVVLQELD 360

************************************************************

C198_mcr-3.41 SEIAQMKGDKLVGFHLIGSHGPTYYKRYPDAHRQFTPDCPRSDIENCTDEELTNTYDNTI 420

172_mcr-3.3 SEIAQMKGDKLVGFHLIGSHGPTYYKRYPDAHRQFTPDCPRSDIENCTDEELTNTYDNTI 420

************************************************************

C198_mcr-3.41 RYTDFVI**G**EMIAKLKTYEDKYNTALLYVSDHGESLGALGLYLHGTPYKFAPDDQTRVPMQ 480

172_mcr-3.3 RYTDFVI**A**EMIAKLKTYEDKYNTALLYVSDHGESLGALGLYLHGTPYKFAPDDQTRVPMQ 480

*******.****************************************************

C198_mcr-3.41 VWMSPGFI**T**EKGMNMECLQKNAAANRYSHDNIFSSVLGIWDVKTAIYEQELDIFKQCRNN 540

172_mcr-3.3 VWMSPGFI**K**EKGMNMECLQKNAAANRYSHDNIFSSVLGIWDVKTAIYEQELDIFKQCRNN 540

********.***************************************************

**Pairwise alignment between *E. coli* carrying MCR-4 and *A. veronii* carrying a novel MCR-4.1**

E.coli_ECCTRSRTH07_mcr-3.4 MPSLIKIKIVPLMFFLALYFAFMLNWRGVLHFYEILYKLEDFKFGFAISLPILLVAALNF 60

C198_mcr-3.41 MPSLIKIKIVPLMFFLALYFAFMLNWRGVLHFYEILYKLEDFKFGFAISLPILLVAALNF 60

************************************************************

E.coli_ECCTRSRTH07_mcr-3.4 VFVPFSIRYLIKPFFALLIALSAIVSYTMMKYRVLFDQNMIQNIFETNQNEALAYLSLPI 120

C198_mcr-3.41 VFVPFSIRYLIKPFFALLIALSAIVSYTMMKYRVLFDQNMIQNIFETNQNEALAYLSLPI 120

************************************************************

E.coli_ECCTRSRTH07_mcr-3.4 I**V**WVTIAGFIPAILLFFVEIEYEEKWFKGILTR**A**LSMFASLIVIAVIAALYYQDYVSVGR 180

C198_mcr-3.41 I**G**WVTIAGFIPAILLFFVEIEYEEKWFKGILTR**T**LSMFASLIVIAVIAALYYQDYVSVGR 180

* *******************************:**************************

E.coli_ECCTRSRTH07_mcr-3.4 NNSNLQREIVPANFVNSTVKYVYNRYLAEPIPFTTLGDDAKRDTNQSKPTLMFLVVGETA 240

C198_mcr-3.41 NNSNLQREIVPANFVNSTVKYVYNRYLAEPIPFTTLGDDAKRDTNQSKPTLMFLVVGETA 240

************************************************************

E.coli_ECCTRSRTH07_mcr-3.4 RGKNFSMNGYEKDTNPFTSKSGGVISFNDVRSCGTATAVSVPCMFSNMGRKEFDDN**R**ARN 300

C198_mcr-3.41 RGKNFSMNGYEKDTNPFTSKSGGVISFNDVRSCGTATAVSVPCMFSNMGRKEFDDN**L**ARN 300

******************************************************** ***

E.coli_ECCTRSRTH07_mcr-3.4 SEGLLDVLQKTG**I**SIFWKENDGGCKGVCDRVPNIEI**E**PKD**H**PKFCDKNTCYDEVVLQ**D**LD 360

C198_mcr-3.41 SEGLLDVLQKTG**V**SIFWKENDGGCKGVCDRVPNIEI**K**PKD**Y**PKFCDKNTCYDEVVLQ**E**LD 360

************:***********************:***:****************:**

E.coli_ECCTRSRTH07_mcr-3.4 SEIAQMKGDKLV**V**FHLIGSHGPTYYKRYPDAHRQFTPDCPRSDIENCTDEELTNTYDNTI 420

C198_mcr-3.41 SEIAQMKGDKLV**G**FHLIGSHGPTYYKRYPDAHRQFTPDCPRSDIENCTDEELTNTYDNTI 420

************ ***********************************************

E.coli_ECCTRSRTH07_mcr-3.4 RYTDFVIGEMIAKLKTYEDKYNTALLYVSDHGESLGALGLYLHGTPY**Q**FAPDDQTRVPMQ 480

C198_mcr-3.41 RYTDFVIGEMIAKLKTYEDKYNTALLYVSDHGESLGALGLYLHGTPY**K**FAPDDQTRVPMQ 480

***********************************************:************

E.coli_ECCTRSRTH07_mcr-3.4 VWMSPGF**TK**EKG**VD**M**A**CLQ**QK**AA**DT**RYSHDNIFSSVLGIWDVKT**SV**YE**KG**LDIF**S**QCRN**V** 540

C198_mcr-3.41 VWMSPGF**IT**EKG**MN**M**E**CLQ**KN**AA**AN**RYSHDNIFSSVLGIWDVKT**AI**YE**QE**LDIF**K**QCRN**N** 540

******* .***::* ***::** .*******************::**: ****.****

E.coli_ECCTRSRTH07_mcr-3.4 **Q** 541

C198_mcr-3.41 - 540

**Alignment of *mcr-3-like* sequence among strains C198, 126-14, 172, and HX3**

C198_mcr-3-like MFSAVRIKVVPFVLLLALVFAFLLNWPVLLHFYDILSNIEHFKIGFVVSIPFLLVAALNF 60

HX3_mcr-3-like MFSAVRIKVVPFVLLLALVFAFLLNWPVLLHFYDILSNIEHFKIGFVVSIPFLLVAALNF 60

126-14_mcr-3-like MFSAVRIKVVPFVLLLALVFAFLLNWPVLLHFYDILSNIEHFKIGFVVSIPFLLVAALNF 60

172_mcr-3-like MFSAVRIKVVPFVLLLALVFAFLLNWPVLLHFYDILSNIEHFKIGFVVSIPFLLVAALNF 60

************************************************************

C198_mcr-3-like VFMPFSIRFLMKPFFAFLFVTGSIASYTMMKYRVLFDGDMIQNIFETNQSEAFAYVNAPI 120

HX3_mcr-3-like VFMPFSIRFLMKPFFAFLFVTGSIASYTMMKYRVLFDGDMIQNIFETNQSEAFAYVNAPI 120

126-14_mcr-3-like VFMPFSIRFLMKPFFAFLFVTGSIASYTMMKYRVLFDGDMIQNIFETNQSEAFAYVNAPI 120

172_mcr-3-like VFMPFSIRFLMKPFFAFLFVTGSIASYTMMKYRVLFDGDMIQNIFETNQSEAFAYVNAPI 120

************************************************************

C198_mcr-3-like IIWVILTGLLPAALIFFVKIEYASTWYKGIAQRLLSMFFSLVIVGIIAALYYQDYASIGR 180

HX3_mcr-3-like IIWVILTGLLPAALIFFVKIEYASTWYKGIAQRLLSMFFSLVIVGIIAALYYQDYASIGR 180

126-14_mcr-3-like IIWVILTGLLPAALIFFVKIEYASTWYKGIAQRLLSMFFSLVIVGIIAALYYQDYASIGR 180

172_mcr-3-like IIWVILTGLLPAALIFFVKIEYASTWYKGIAQRLLSMFFSLVIVGIIAALYYQDYASIGR 180

************************************************************

C198_mcr-3-like NNQTLNREIVPANFMYSTSKYLYRRYMAEPIPFVTLGDDATRVTKKDKPTLMFLVVGETA 240

HX3_mcr-3-like NNQTLNREIVPANFMYSTSKYLYRRYMAEPIPFVTLGDDATRVTKKDKPTLMFLVVGETA 240

126-14_mcr-3-like NNQTLNREIVPANFMYSTSKYLYRRYMAEPIPFVTLGDDATRVTKKDKPTLMFLVVGETA 240

172_mcr-3-like NNQTLNREIVPANFMYSTSKYLYRRYMAEPIPFVTLGDDATRVTKKDKPTLMFLVVGETA 240

************************************************************

C198_mcr-3-like RGKNFSMNGYEKDTNPFTSKSGGVISFNDVRS**C**GTATAVSVPCMFSNMGRKEFDDNRARN 300

HX3_mcr-3-like RGKNFSMNGYEKDTNPFTSKSGGVISFNDVRS**C**GTATAVSVPCMFSNMGRKEFDDNRARN 300

126-14_mcr-3-like RGKNFSMNGYEKDTNPFTSKSGGVISFNDVRS**C**GTATAVSVPCMFSNMGRKEFDDNRARN 300

172_mcr-3-like RGKNFSMNGYEKDTNPFTSKSGGVISFNDVRS**Y**GTATAVSVPCMFSNMGRKEFDDNRARN 300

******************************** ***************************

C198_mcr-3-like SEGLLDVLQKTGISIFWKENDGGCKGVCDRVPNIEIEPKDHPKFCDKNTCYDEVVLQDLD 360

HX3_mcr-3-like SEGLLDVLQKTGISIFWKENDGGCKGVCDRVPNIEIEPKDHPKFCDKNTCYDEVVLQDLD 360

126-14_mcr-3-like SEGLLDVLQKTGISIFWKENDGGCKGVCDRVPNIEIEPKDHPKFCDKNTCYDEVVLQDLD 360

172_mcr-3-like SEGLLDVLQKTGISIFWKENDGGCKGVCDRVPNIEIEPKDHPKFCDKNTCYDEVVLQDLD 360

************************************************************

C198_mcr-3-like SEIAQMKGDKLVGFHLIGSHGPTYYKRYPDAHRQFTPDCPRSDIENCTDEELTNTYDNTI 420

HX3_mcr-3-like SEIAQMKGDKLVGFHLIGSHGPTYYKRYPDAHRQFTPDCPRSDIENCTDEELTNTYDNTI 420

126-14_mcr-3-like SEIAQMKGDKLVGFHLIGSHGPTYYKRYPDAHRQFTPDCPRSDIENCTDEELTNTYDNTI 420

172_mcr-3-like SEIAQMKGDKLVGFHLIGSHGPTYYKRYPDAHRQFTPDCPRSDIENCTDEELTNTYDNTI 420

************************************************************

C198_mcr-3-like RYTDFVIGEMIAKLKTYEDKYNTALLYVSDHGESLGALGLYLHGTPYKFAPDDQTRVPMQ 480

HX3_mcr-3-like RYTDFVIGEMIAKLKTYEDKYNTALLYVSDHGESLGALGLYLHGTPYKFAPDDQTRVPMQ 480

126-14_mcr-3-like RYTDFVIGEMIAKLKTYEDKYNTALLYVSDHGESLGALGLYLHGTPYKFAPDDQTRVPMQ 480

172_mcr-3-like RYTDFVIGEMIAKLKTYEDKYNTALLYVSDHGESLGALGLYLHGTPYKFAPDDQTRVPMQ 480

************************************************************

C198_mcr-3-like VWMSPGFTKEKGVDMACLQQKAADTRYSHDNIFSSVLGIWDVKTSVYEKGLDIFSQCRNV 540

HX3_mcr-3-like VWMSPGFTKEKGVDMACLQQKAADTRYSHDNIFSSVLGIWDVKTSVYEKGLDIFSQCRNV 540

126-14_mcr-3-like VWMSPGFTKEKGVDMACLQQKAADTRYSHDNIFSSVLGIWDVKTSVYEKGLDIFSQCRNV 540

172_mcr-3-like VWMSPGFTKEKGVDMACLQQKAADTRYSHDNIFSSVLGIWDVKTSVYEKGLDIFSQCRNV 540

************************************************************

C198_mcr-3-like Q 541

HX3_mcr-3-like Q 541

126-14_mcr-3-like Q 541

172_mcr-3-like Q 541

*
