# Supplementary material for: Genomic Analysis of Aeromonas veronii C198, a Novel Mcr-3.41-Harboring Isolate from a Patient with Septicemia in Thailand
Source: Pathogens. 2020 Dec 9;9(12):1031. doi: 10.3390/pathogens9121031 (PMC7763265; doi:10.3390/pathogens9121031)
Supplement: Supplementary file 1 [file pathogens-09-01031-s001.zip › Supplementary files/Supplementary file 2-The mcr-3 protein sequences used for phylogenetic tree construction.docx]

**Supplementary Table**. The mcr-3 protein sequences used for phylogenetic tree construction

| **No.** | **mcr-3 variants** | **Accession number** | **Species** |
| --- | --- | --- | --- |
| 1 | mcr-3.1 | BBG40253 | *Escherichia coli* |
| 2 | mcr-3.2 | AUF81229 | *Escherichia coli* |
| 3 | mcr-3.3 | QGW97965 | *Aeromonas veronii* |
| 4 | mcr-3.4 | TNK33736 | *Escherichia coli* |
| 5 | mcr-3.5 | QKY84243 | *Escherichia coli* |
| 6 | mcr-3.6 | BBT06325 | *Aeromonas hydrophila* |
| 7 | mcr-3.7 | AWM63398 | *Escherichia coli* |
| 8 | mcr-3.8 | AST36143 | *Aeromonas hydrophila* |
| 9 | mcr-3.9 | AST36144 | *Aeromonas hydrophila* |
| 10 | mcr-3.10 | WP_099982820 | *Aeromonas caviae* |
| 11 | mcr-3.11 | AWU78652 | *Klebsiella pneumoniae* |
| 12 | mcr-3.12 | WP_109545070 | *Escherichia coli* |
| 13 | mcr-3.13 | WP_111273842 | *Aeromonas caviae* |
| 14 | mcr-3.14 | BBQ51531 | *Aeromonas veronii* |
| 15 | mcr-3.15 | WP_111273844 | *Aeromonas media* |
| 16 | mcr-3.16 | WP_111273845 | *Aeromonas salmonicida* |
| 17 | mcr-3.17 | WP_111273846 | *Aeromonas sp.* |
| 18 | mcr-3.18 | BBT52044 | *Aeromonas caviae* |
| 19 | mcr-3.19 | AYN87874 | *Escherichia coli* |
| 20 | mcr-3.20 | QBC75463 | *Escherichia coli* |
| 21 | mcr-3.21 | QFU73659 | *Klebsiella pneumoniae* |
| 22 | mcr-3.22 | QDJ80325 | *Klebsiella pneumoniae* |
| 23 | mcr-3.23 | WP_094313523 | *Klebsiella pneumoniae* |
| 24 | mcr-3.24 | WP_094321595 | Enterobacteriaceae |
| 25 | mcr-3.25 | WP_103252528 | *Aeromonas veronii* |
| 26 | mcr-3.26 | WP_140423331 | *Klebsiella pneumoniae* |
| 27 | mcr-3.27 | AXS68550 | *Aeromonas hydrophila* |
| 28 | mcr-3.28 | WP_150823496 | *Klebsiella pneumoniae* |
| 29 | mcr-3.29 | WP_136512112 | *Escherichia coli* |
| 30 | mcr-3.30 | WP_140423332 | *Aeromonas veronii* |
| 31 | mcr-3.31 | WP_188331890 | Uncultured bacterium |
| 32 | mcr-3.32 | WP_188331891 | Uncultured bacterium |
| 33 | mcr-3.33 | WP_188331892 | *Aeromonas jandaei* |
| 34 | mcr-3.34 | WP_188331893 | *Aeromonas jandaei* |
| 35 | mcr-3.35 | WP_188331894 | *Aeromonas jandaei* |
| 36 | mcr-3.36 | WP_188331895 | *Aeromonas jandaei* |
| 37 | mcr-3.37 | WP_188331896 | *Aeromonas veronii* |
| 38 | mcr-3.38 | WP_039039919 | *Aeromonas caviae* |
| 39 | mcr-3.39 | QNG62238 | *Escherichia coli* |
| 40 | mcr-3.40 | WP_188331897 | *Klebsiella pneumoniae* |
| 41 | mcr-3.41 | MBA2799562.1 | *Aeromonas veronii* |
